# Low-dose metformin in the brain: A focused review of its novel mechanism of glucose lowering in type 2 diabetes

**DOI:** 10.17305/bb.2026.14097

**Published:** 2026-04-07

**Authors:** Zain Ahmad, Saghir Akhtar

**Affiliations:** 1 College of Medicine, QU Health, Qatar University, Doha, Qatar

**Keywords:** Metformin, type 2 diabetes mellitus, glucose homeostasis, hypothalamus, ventromedial hypothalamus, Rap1, SF1 neurons, AMPK, GLP-1, central glucose regulation

## Abstract

Metformin remains the first-line pharmacological therapy for type 2 diabetes mellitus (T2DM). While its glucose-lowering effects have primarily been attributed to peripheral actions, evidence indicates that it also crosses the blood–brain barrier (BBB) and can exert anti-diabetic effects from within the central nervous system (CNS). This focused review discusses metformin’s central actions and how they might integrate with established peripheral mechanisms of glucose-lowering. We synthesized recent mechanistic studies conducted in murine models using systemic and intracerebroventricular metformin administration, brain-specific genetic loss- and gain-of-function approaches, neuronal activation mapping, electrophysiology, and pharmacological interrogation of autonomic and metabolic pathways. The evidence reviewed indicates that clinically relevant low concentrations of metformin in the brain engage a ventromedial hypothalamus (VMH)–steroidogenic factor 1 (SF1) neuron–Ras-proximate-1 (Rap1) signaling axis, which is essential for its glucose lowering action. Notably, metformin inhibits Rap1 in VMH SF1 neurons to reduce hyperglycemia. This central mechanism appears specific to metformin and is not shared by other approved antidiabetic agents. Current data also imply that the CNS effects of metformin operate in conjunction with peripheral pathways including hepatic AMP-activated protein kinase (AMPK) signaling and gut-derived glucagon-like peptide-1 (GLP-1) secretion, to produce its overall metabolic effect. Collectively, these findings support a model in which metformin lowers glucose through an orchestrated integration of a central Rap-1 dependent mechanism and peripheral metabolic actions. Additionally, the recognition of a central component to metformin’s pharmacology expands the mechanistic framework of this cornerstone therapy and may inform future therapeutic strategies targeting integrated brain–metabolic pathways in T2DM.

## Introduction

Type 2 diabetes mellitus (T2DM) is a chronic metabolic disorder characterized by insulin resistance and progressive beta-cell dysfunction. It is associated with long-term complications of chronic hyperglycemia, including cardiovascular disease, peripheral neuropathy, nephropathy, and retinopathy [1–5]. In clinical practice, metformin, in conjunction with diet and exercise, remains the preferred pharmacological agent for managing T2DM. This biguanide is administered orally at total daily doses ranging from 500–2550 mg [6]. At clinically relevant doses, metformin decreases hepatic glucose production and intestinal glucose absorption while enhancing insulin sensitivity by increasing peripheral glucose uptake and utilization [7–9]. These effects are contingent upon transporter-mediated tissue uptake and intracellular modifications in mitochondrial metabolism and energy-sensing pathways, including AMPK-dependent signaling cascades [7–9]. Following oral administration, metformin enters hepatocytes via organic cation transporter-1 (OCT1) and accumulates in mitochondria due to its positive charge and membrane potentials. Within hepatocytes, it inhibits mitochondrial complex I, leading to reduced ATP production and increased cellular AMP:ATP and ADP:ATP ratios. This energy shift activates AMP-activated protein kinase (AMPK), a critical regulator of metabolic homeostasis, and directly suppresses gluconeogenic pathways by inhibiting fructose-1,6-bisphosphatase and adenylate cyclase, thereby lowering cAMP levels [7–9]. Activated AMPK further diminishes lipid synthesis through the phosphorylation of acetyl-CoA carboxylase, promotes fatty acid oxidation, reduces hepatic lipid accumulation, and enhances hepatic insulin sensitivity, collectively improving glycemic control in T2DM [7–9]. Furthermore, metformin can stimulate GLP-1 secretion by directly activating intestinal L-cells through an AMPK-dependent mechanism, further regulating glucose homeostasis [7, 10–12].

### Central regulation of glucose homeostasis

Metformin is also known to cross the blood-brain barrier (BBB), albeit at relatively low concentrations, raising the question of whether it exerts anti-hyperglycemic effects through central nervous system (CNS) mechanisms [13]. Evidence indicates that glucose homeostasis can be regulated centrally (see Table 1A for summary) [14–19]. Initial studies demonstrated that the brain directly regulates glucose homeostasis through hypothalamic nutrient-sensing mechanisms. For instance, Obici et al. showed that central administration of the long-chain fatty acid (LCFA) oleic acid suppressed hepatic glucose production and food intake, effects not replicated by the short-chain fatty acid octanoic acid. This response required hypothalamic ATP-sensitive potassium (KATP) channel activity, indicating a specificity for long-chain lipid sensing [14]. Pocai et al. extended this framework by demonstrating that KATP channel activation in the mediobasal hypothalamus (MBH) lowers blood glucose primarily by inhibiting hepatic gluconeogenesis. This effect was abolished by blocking hypothalamic KATP channels, and surgical resection of the hepatic vagal branch disrupted the response, delineating a distinct brain-liver circuit [15]. Lam et al. further established that under physiologically relevant conditions, MBH sensing of LCFAs, following their conversion to long-chain fatty acyl-CoA, activates the same KATP channel and vagal efferent pathway to preserve hepatic autoregulation [16]. Importantly, this central lipid-sensing pathway enhances hepatic autoregulation by increasing gluconeogenesis while preventing a significant rise in total glucose production due to a compensatory decrease in glycogenolysis [16]. Collectively, these studies establish that the MBH integrates lipid-derived nutrient signals and translates them into coordinated hepatic glucose regulation via a KATP channel–vagal axis.

**Table 1:** Summary of key studies illustrating A) the role of the brain in regulating glucose homeostasis and B) the central mechanisms of metformin in glucose reduction

**Site / axis**	**Model**	**Species**	**Assay**	**Dose/route**	**Key finding**	**Reference**
**A. Example studies showing the brain as a regulator of glucose homeostasis**			
MBH – hypothalamus–liver axis	Pancreatic clamp + ICV insulin	Rat	Glucose production rate	ICV insulin	Central insulin signaling suppresses hepatic glucose production via hypothalamic pathways.	Obici et al., 2002 [14]
MBH – lipid sensing → vagus → liver	ICV lipid infusion ± KATP blockade	Rat	Hepatic glucose production	ICV oleic acid ± glibenclamide	Hypothalamic LCFA sensing via KATP channels and vagal output restrains glucose production	Lam et al., 2005 [16]
MBH – KATP–vagus axis	ICV diazoxide/SUR1 KO	Mouse/rat	Glucose production and gluconeogenesis	ICV diazoxide/KATP blockade	Activation of hypothalamic KATP channels inhibits hepatic gluconeogenesis; loss of KATP abolishes the effect	Pocai et al., 2005 [15]
**B. Example studies on metformin’s centrally acting mechanisms in glucose lowering**			
Brain→gut vs brain→liver	ICV metformin with vagotomy / sympathectomy	Mouse	OGTT; gastric emptying; hepatic markers	≈30 µg; ICV	Vagal route improves tolerance via slower gastric emptying; sympathetic route can increase gluconeogenic signaling	Jin et al., 2023 [22]
Central (VMH SF1 Rap1)	Forebrain or SF1-specific Rap1 manipulation; metformin challenge	Mouse	GTT; glucose lowering; neuronal activity	Low-dose; ICV and systemic	Low dose metformin effect is dependent on reduced VMH Rap1 activity; constitutively active Rap1 (Rap1V12) blocks the benefit	Lin et al., 2025 [23]
Central	ICV biguanide administration in diabetic models (D-glucose–fed and STZ-treated)	Mouse	Blood glucose measurement; GTT	ICV metformin (central administration; 20 or 30 µg)	ICV metformin significantly lowered blood glucose in diabetic mice, demonstrating a direct central anti-diabetic effect.	Sim YB et al., 2012 [25]
Central (hypothalamus)	Obese diabetic rats treated with metformin	Rat	Food intake; blood glucose; hypothalamic signaling markers (AMPK-related pathways)	Systemic metformin (oral administration; chronic treatment)	Metformin reduced food intake and improved glycemia in obese diabetic rats, associated with altered hypothalamic metabolic signaling.	Lv WS et al., 2012 [24]

**Abbreviations:** AMPK, AMP-activated protein kinase; GTT, glucose tolerance test; ICV, intracerebroventricular; KATP, ATP-sensitive potassium; KO, knockout; LCFA, long-chain fatty acid; MBH, mediobasal hypothalamus; oGTT, oral glucose tolerance test; Rap1, Ras-proximate-1; Rap1V12, constitutively active Rap1 mutant; SF1, steroidogenic factor 1; STZ, streptozotocin; SUR1, sulfonylurea receptor 1; VMH, ventromedial hypothalamus.

In addition to MBH nutrient-sensing mechanisms, other hypothalamic regions have also been implicated in central glucoregulation [14–16]. Notably, the ventromedial hypothalamus (VMH) has been identified as a key coordinator of metabolism, regulating energy balance and hepatic glucose production [20]. Specifically, steroidogenic factor 1 (SF1) neurons, the principal neuronal population within the VMH, have been shown to regulate systemic glucose levels [20]. Independently, Ras-proximate-1 (Rap1) signaling within SF1 neurons also contributes to systemic energy and glucose homeostasis [20]. Rap1, a small GTPase of the Ras superfamily functioning as a molecular switch, influences synaptic plasticity, neuronal polarity, and activity-dependent signaling [20].

Given the demonstrated importance of central pathways in glucose homeostasis, it remains to be determined whether metformin engages a central mechanism, such as the VMH–Rap1 pathway, to elicit its glucose-lowering effects. Recent studies have provided significant insights into the central glucose-lowering actions of metformin and their potential mechanisms [21–24] (see also summary in Table 1B).

### Evidence for central actions of metformin

A study by Jin et al. provided evidence that metformin acts within the brain to regulate systemic glucose levels [22]. Utilizing high-dose (30 µg) intracerebroventricular (ICV) administration combined with selective vagotomy and sympathectomy, they demonstrated that central metformin influences glycemic control through two opposing autonomic pathways: a beneficial brain-gut (vagal) pathway that enhances oral glucose tolerance and a regulatory brain-liver (sympathetic) pathway that increases hepatic glucose production [22]. Previous CNS-focused research similarly indicated that centrally administered biguanides can reduce blood glucose levels in murine models [25], supporting the notion that central metformin exposure produces glucose-lowering effects. However, these studies have notable limitations. First, the high CNS concentrations utilized cannot typically be achieved through oral metformin administration, raising questions about the relevance of glucose-lowering effects observed with direct high-dose ICV delivery in clinical practice. Second, these studies do not elucidate the intracellular pathways through which central metformin acts, leaving mechanistic insights lacking.

### A novel Rap1-dependent mechanism of low dose metformin

To address these limitations, Lin et al. recently investigated whether low, physiologically relevant brain concentrations of metformin could influence systemic glucose regulation [23]. Systemic administration of 150 mg/kg metformin resulted in low hypothalamic levels of 4.6 µM after four hours, a concentration sufficient to affect neuronal excitability [13]. At this dose, systemic metformin produced a glycemia-lowering response in control mice. In contrast, CNS-specific Rap1-deficient mice exhibited no glucose-lowering effect following the same treatment [23]. This finding suggests that central Rap1 signaling is essential for the glucose-lowering response to low-dose metformin. Given that systemic metformin required central Rap1 to lower blood glucose, the authors further investigated whether metformin directly activates VMH SF1 neurons.

The researchers administered the drug directly into the brain via ICV injection (1–5 µg), and four hours later, brain tissue was processed to assess VMH activation using c-Fos [23]. c-Fos is a classic immediate-early gene marker rapidly expressed in neurons following synaptic activation and is widely used to map engaged neuronal populations in neuroendocrine circuits [26]. c-Fos induction was observed in VMH SF1 neurons, indicating a brain-specific effect of low-dose metformin [23]. However, the activation of VMH neurons after ICV metformin does not necessarily confirm that metformin acts directly on SF1 neurons, as this response could arise from upstream hypothalamic inputs.

To determine whether metformin acts directly on VMH SF1 neurons, the authors performed *ex vivo* electrophysiology using VMH brain slices. They demonstrated that metformin depolarizes the resting membrane potential of SF1 neurons, increasing their firing frequency, thereby indicating that metformin directly enhances the excitability of these neurons [23]. To assess whether this electrophysiological response requires Rap1 signaling, the authors repeated the recordings in SF1-specific Rap1 knockout mice. Under these conditions, metformin failed to depolarize SF1 neurons lacking Rap1, and their firing frequency showed no increase, suggesting that Rap1 is required for metformin to excite SF1 neurons. This finding implies that metformin directly engages the VMH–SF1–Rap1 pathway, identifying this circuit as a potential central mediator of metformin’s glucose-lowering mechanism [23].

Lin et al. subsequently examined whether Rap1 dependence was specific to metformin or shared across the glucose-lowering effects of other clinically used antidiabetic drugs. Using Rap1 loss-of-function models, they administered several other antidiabetic drugs, including exendin-4 (a GLP-1 receptor agonist), rosiglitazone (a thiazolidinedione), glibenclamide (a sulfonylurea), dapagliflozin (a SGLT2 inhibitor), and Humulin R (a short-acting insulin). These treatments produced normal glucose-lowering responses despite the absence of Rap1 in the hypothalamus of the mice [23]. In contrast, the glucose-lowering effect of low-dose metformin was markedly reduced, indicating that metformin uniquely relies on a Rap1-dependent VMH pathway, whereas the other antidiabetic drugs tested do not appear to utilize the same pathway for their glucose-lowering effects [23]. To establish directionality for Rap1 signaling, they employed genetic gain-of-function approaches [23]. Constitutively active Rap1 in the VMH diminished metformin’s ability to lower glucose levels and blunted improvements in glucose tolerance [23]. Collectively, these findings suggest that metformin’s centrally mediated glycemia-lowering response depends on inhibiting Rap1 signaling in VMH SF1 neurons, a mechanism not shared by other common antidiabetic drugs (see also Table 1B).

### Possible pathways linking metformin’s central actions to the periphery for glycemic control

While Lin et al. [23] identified a key hypothalamic Rap-1-dependent mechanism for metformin’s glycemia-lowering action, the precise manner in which this central mechanism communicates with peripheral organs involved in glucose homeostasis remains unclear. The brain continuously monitors systemic glucose levels by transmitting glycemic information from peripheral glucose-sensing neurons, such as those in the portal-mesenteric vein, to the hypothalamus [27–29]. Vagal and spinal afferents convey information to hypothalamic circuits, where glucose-sensing neurons in the dorsomedial hypothalamus, VMN, and lateral hypothalamus respond to regulate glucose homeostasis [27, 29]. The arcuate nucleus integrates these signals, with AgRP and POMC neurons encoding opposing states of energy availability and projecting to the paraventricular nucleus, which functions as the principal output center coordinating autonomic and neuroendocrine signals to peripheral glucose effector organs. Meanwhile, the VMN drives counterregulatory hormonal and autonomic responses during hypoglycemia (for a review, see [27, 28]). Disruption of these pathways, including impaired KATP channel-dependent feedback onto AgRP neurons, may underlie defective glucose regulation in type 2 diabetes, although the mechanisms by which agents such as metformin engage these central circuits remain to be elucidated [27].

Although Lin et al. [23] did not investigate the downstream efferent autonomic pathways beyond Rap1 that mediate the central glucose-lowering effects of metformin, the mechanisms involved are likely dose-dependent. Higher doses have been shown to produce partial glucose lowering even in Rap1-deficient models, implying the involvement of Rap1-independent pathways at elevated doses (see also Table 1B). Current studies suggest that low-dose metformin likely activates the beneficial vagal stimulatory brain-gut pathway for glucose-lowering [23], whereas high centrally administered doses are known to additionally activate a counter-regulatory sympathetic (brain–liver) route that increases hepatic glucose production [22] (see also Figure 1). Furthermore, low-dose metformin selectively activates the lysosomal PEN2-vATPase-AMPK signaling complex in the liver and intestine, exerting its anti-diabetic effects [30]. Given that Rap1 is also thought to localize to the lysosomal membrane [31, 32], these findings raise the intriguing possibility that Rap1 and lysosomal AMPK signaling converge within hypothalamic circuits, which are exposed to low metformin concentrations, thereby coordinating its glucose-lowering effects.

**Figure 1.**
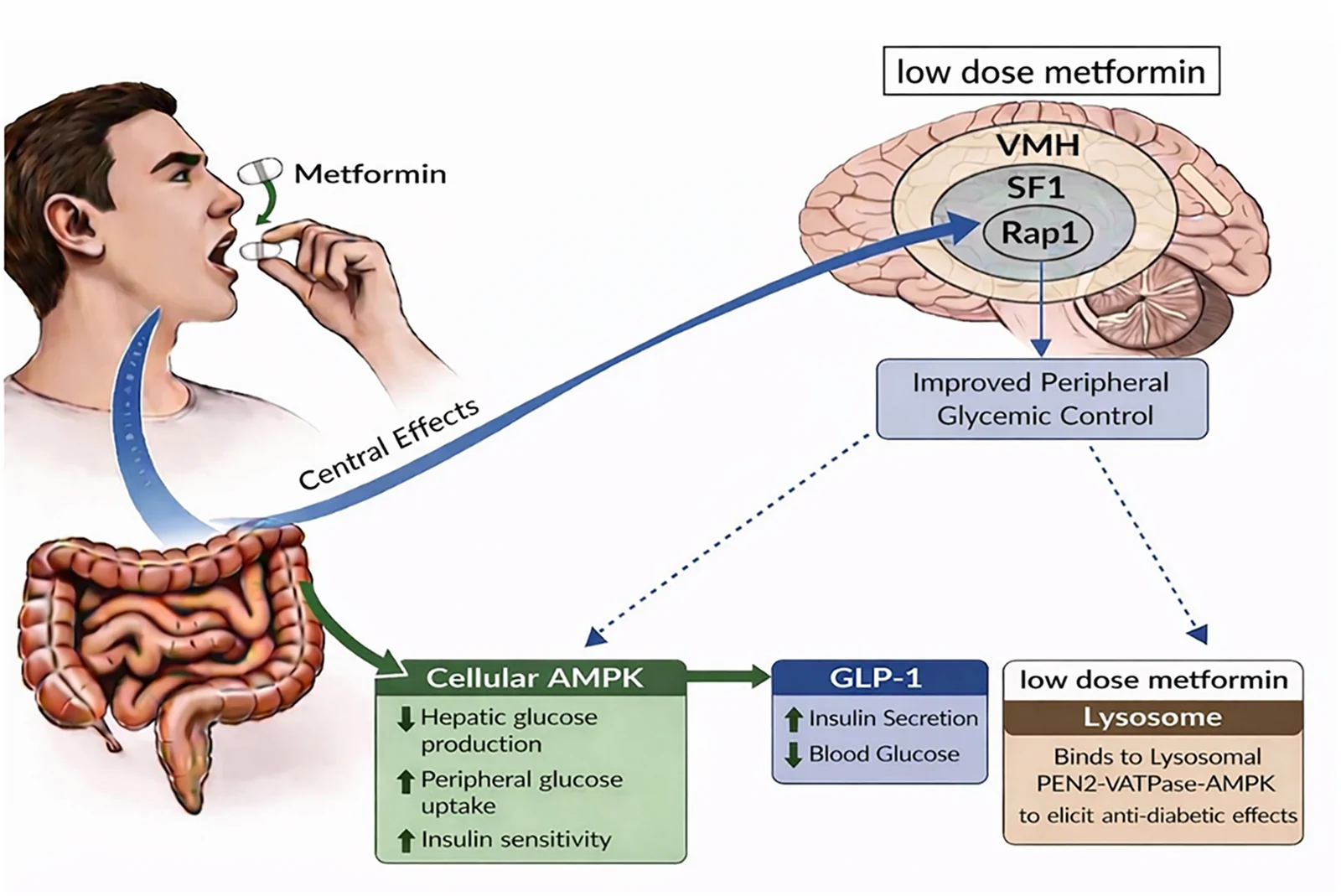
**Proposed model of metformin action integrating central Rap1-dependent and peripheral Rap1-independent mechanisms**. Upon oral administration, metformin enters the bloodstream and crosses the blood-brain barrier, enabling biologically relevant concentrations to reach the central nervous system. At these concentrations, metformin is thought to inhibit Rap1 signaling in SF1 neurons of the VMH, which increases VMH neuronal activity and activates a brain-mediated glucose-lowering pathway. In this framework, the central Rap1-dependent mechanism enhances downstream autonomic output, improving oral glucose tolerance and contributing to systemic glycemic control. Concurrently, metformin exerts established peripheral Rap1-independent effects through an AMPK-dependent pathway, including the secretion of GLP-1. Enhanced GLP-1 signaling promotes insulin secretion and reduces blood glucose levels, while AMPK-dependent actions suppress hepatic glucose production, decrease intestinal glucose absorption, increase peripheral glucose uptake/utilization, and enhance insulin sensitivity. Additionally, low-dose peripheral metformin can bind to the lysosomal PEN2-vATPase-AMPK complex in the liver and intestine, eliciting peripheral anti-diabetic effects. This figure illustrates metformin as a drug whose overall glucose-lowering effect may result from the coordinated integration of a central VMH-SF1-Rap1 pathway with parallel peripheral metabolic mechanisms. Central GLP-1 actions are not depicted; although GLP-1 can lower glucose through brain circuits, available evidence suggests that this effect is mechanistically distinct from the Rap1-dependent VMH pathway proposed for low-dose metformin. As the precise downstream autonomic relay circuits linking VMH Rap1 inhibition to peripheral glucose effector organs are not yet fully defined, this schematic represents a proposed model. Accordingly, dotted lines indicate hypothetical pathways, while solid lines denote experimentally established mechanisms. *This figure was generated with assistance of ChatGPT (OpenAI, San Francisco, CA, USA) and significantly modified by the authors.* **Abbreviations:** AMPK, AMP-activated protein kinase; GLP-1, glucagon-like peptide-1; PEN2, presenilin enhancer 2; Rap1, Ras-proximate-1; SF1, steroidogenic factor 1; vATPase, vacuolar-type H+-ATPase; VMH, ventromedial hypothalamus.

Another unresolved question pertains to the interplay between metformin and central GLP-1 signaling. Lin et al. demonstrated that exendin-4, a GLP-1 receptor agonist, retained its glucose-lowering efficacy in brain-specific Rap1-deficient mice, indicating that GLP-1 receptor agonists act independently of Rap1 and through mechanisms distinct from metformin [23]. By employing intraperitoneal rather than oral metformin administration, the authors avoided the confounding peripheral GLP-1 secretion associated with enteral delivery [7, 10–12]. This distinction is crucial, as GLP-1 exerts both peripheral and central glucoregulatory actions. Within the CNS, GLP-1 is synthesized by neurons in the nucleus tractus solitarius and projects to multiple hypothalamic regions, including the dorsomedial hypothalamus (DMH) [33]. Recent *in vivo* evidence has identified GLP-1 receptor-expressing neurons in the DMH as glucose-sensing, with their activation sufficient to reduce blood glucose levels [33]. Huang et al. further demonstrated that DMH GLP-1 signaling is both necessary and sufficient for glucose lowering, operating through a hypothalamus–brainstem–pancreas axis that enhances insulin secretion via cAMP-PKA–dependent inhibition of potassium currents, leading to neuronal depolarization and activation [33]. Collectively, these findings support a model in which central GLP-1 signaling engages hypothalamic glucose-sensing circuits to exert systemic glucoregulatory control.

In this context, the question arises as to whether centrally acting metformin could recruit CNS GLP-1 signaling as part of its mechanism. Evidence from Lin et al. [23] suggests otherwise: centrally administered metformin did not produce further glucose lowering in brain-specific Rap1-deficient mice, indicating that its central effects are fully accounted for by Rap1-dependent signaling and do not additionally engage GLP-1 pathways within the CNS, although further study is warranted. Thus, current evidence supports Rap1 as a central requirement for the action of low-dose metformin, while the downstream autonomic routes and the interaction between central Rap1 signaling and peripheral GLP-1 effects remain unresolved.

## Conclusion

Accumulating evidence indicates that metformin traverses the blood–brain barrier and exerts clinically relevant central nervous system effects through a Rap1-dependent mechanism in SF1-expressing neurons of the VMH [23]. These actions operate in concert with its well-characterized peripheral effects on hepatic glucose production and insulin sensitivity (Figure 1; Table 1). Beyond its established role in suppressing hepatic gluconeogenesis, metformin appears to modulate hypothalamic circuits governing appetite regulation and autonomic outflow [34–37]. By modulating brain–liver signaling, metformin may suppress sympathetic drive to the liver and attenuate fasting glucose production independently of direct peripheral hepatocellular effects. Central mechanisms may also contribute to its weight-neutral or modest weight-reducing profile by improving hypothalamic insulin and leptin sensitivity and dampening orexigenic signaling pathways [34–37]. These neuro-metabolic effects provide a plausible explanation for the low risk of hypoglycemia observed with metformin monotherapy and may partially account for interindividual variability in glycemic response. Although the translational significance of these CNS actions requires further clinical validation, recognizing a central component to metformin’s pharmacology expands its mechanistic framework and may inform future strategies targeting integrated brain–metabolic pathways in type 2 diabetes and related disorders. Ultimately, central Rap1 signaling may represent a key component of metformin’s glucose-lowering action and provide a new framework for understanding its integrated central and peripheral effects.

## Data Availability

No new data were created or analyzed in this study as it is a narrative review. Data sharing is not applicable to this article.
